# XB130 Deficiency Affects Tracheal Epithelial Differentiation during Airway Repair

**DOI:** 10.1371/journal.pone.0108952

**Published:** 2014-10-01

**Authors:** Jinbo Zhao, Yingchun Wang, Andrew Wakeham, Zhenyue Hao, Hiroaki Toba, Xiaohui Bai, Shaf Keshavjee, Tak W. Mak, Mingyao Liu

**Affiliations:** 1 Latner Thoracic Surgery Research Laboratories, Toronto General Research Institute, University Health Network, Toronto, Ontario, Canada; 2 Department of Thoracic Surgery, Tangdu Hospital, Forth Military Medical University, Xi’an, Shaanxi, China; 3 Advanced Medical Discovery Institute, Faculty of Medicine, University of Toronto, Toronto, Ontario, Canada; 4 Department of Surgery, Faculty of Medicine, University of Toronto, Toronto, Ontario, Canada; 5 Institute of Medical Science, Faculty of Medicine, University of Toronto, Toronto, Ontario, Canada; University of Colorado, Denver, United States of America

## Abstract

The repair and regeneration of airway epithelium is important for maintaining homeostasis of the respiratory system. XB130 is an adaptor protein involved in the regulation of cell proliferation, survival and migration. In the human trachea, XB130 is expressed on the apical site of ciliated epithelial cells. We hypothesize that XB130 may play a role in epithelial repair and regeneration after injury. *Xb130* knockout (KO) mice were generated, and a mouse isogenic tracheal transplantation model was used. Adult *Xb130* KO mice did not show any significant anatomical and physiological phenotypes in comparison with their wild type (WT) littermates. The tracheal epithelium in *Xb130* KO mice, however, was significantly thicker than that in WT mice. Severe ischemic epithelial injury was observed immediately after the tracheal transplantation, which was followed by epithelial cell flattening, proliferation and differentiation. No significant differences were observed in terms of initial airway injury and apoptosis. However, at Day 10 after transplantation, the epithelial layer was significantly thicker in *Xb130* KO mice, and associated with greater proliferative (Ki67+) and basal (CK5+) cells, as well as thickening of the connective tissue and fibroblast layer between the epithelium and tracheal cartilages. These results suggest that XB130 is involved in the regulation of airway epithelial differentiation, especially during airway repair after injury.

## Introduction

Epithelial cells in the lung act as the front line of defense against various infectious and noxious substances inhaled from the air. Specifically, the epithelium of the respiratory tract is subject to various chemical, physical, environmental and inflammatory insults. The severity of injury varies from temporary induction of surface epithelium permeability, to cell death and denudation of the epithelial lining. However, under normal physiological conditions, proper repair restores the structure and function of the lung [1]. Highly differentiated epithelial cells exert various physiological functions at different pulmonary compartments within the respiratory system [2]. For example, pseudo-stratified epithelium containing tall columnar ciliated cells, secretory cells (Clara cells and goblet cells) and basal cells line the upper respiratory tree [2].

The mouse trachea has similar cell types, tissue structure and inner diameter when compared with the human small airway [3]. In mice, allogeneic heterotypic tracheal transplantation induces similar pathological changes as seen in bronchiolitis obliterans, a form of chronic allograft dysfunction after human lung transplantation [4]. Therefore, this model has been widely used to study the mechanisms of bronchiolitis obliterans syndrome[5]–[8]. In addition, the isogenic heterotypic tracheal transplantation model has been used to study the cellular and molecular mechanisms involved in airway epithelial repair and regeneration[9]–[11]. After transplantation, tracheal airway damage, death and shedding of epithelial cells occur, while surviving basal cells remain attached to the basal lamina. Migration of neighboring basal cells cover the wound [2], followed by cell proliferation, active mitosis, squamous metaplasia and, lastly, progressive re-differentiation of epithelial cells [12]. In summary, migration, proliferation and differentiation are three common steps involved in the repair and regeneration of epithelia in the lung.

XB130 (also called AFAP1L2, for actin filament associated protein 1 like 2) is a newly discovered adaptor protein [13]. XB130 is involved in the regulation of cell proliferation, survival and migration, through its binding with p85α, the regulatory subunit of PI3K, and subsequent activation of PI3K/Akt related signaling[14]–[16]. Microarray and bioinformatics studies have shown that stably knockdown *Xb130* using shRNA significantly changed multiple gene expression [15]. Ingenuity pathway analysis has shown that the top molecular and cellular functions of XB130-related genes are cellular growth and proliferation, and cell cycle [15]. *XB130* could also regulate gene expression through miRNAs [17]. XB130 protein is found mainly in various epithelial cells [15], [18], [19]. Therefore, we hypothesized that XB130 is involved in airway epithelial repair and regeneration. In the present study, *Xb130* knockout mice were generated and used to determine the role of XB130 in airway epithelial repair and regeneration, using the isogenic mouse heterotopic tracheal transplantation model.

## Materials and Methods

### Generation of *Xb130* knockout mice

#### Generation of targeting construct

**Figure 1:**
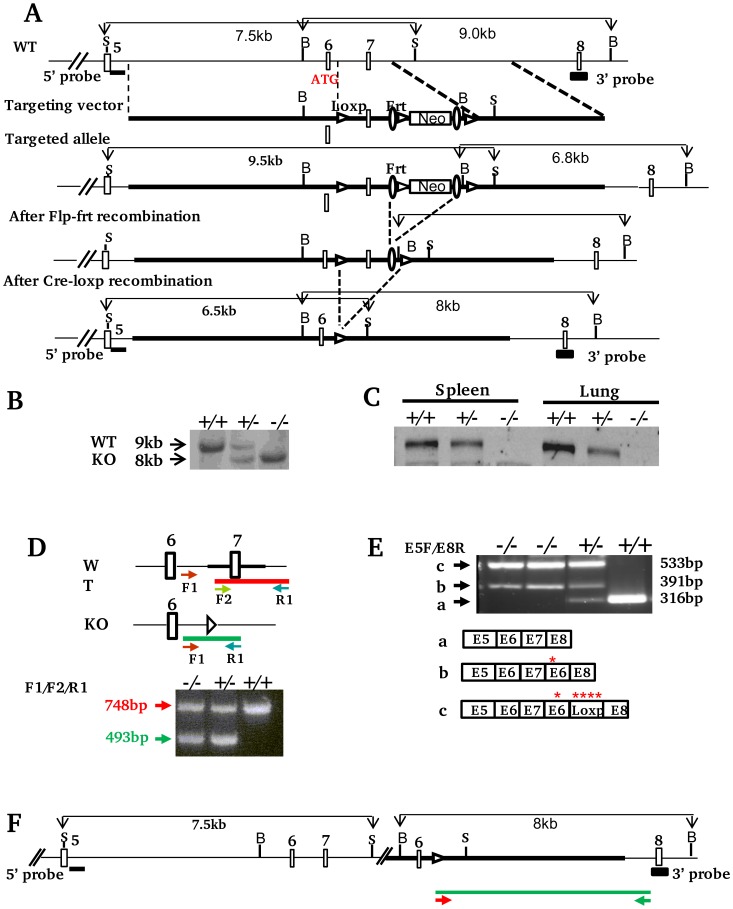
Generation of *Xb130^−/−^* mice. *A. Schematic strategy of xb130 gene targeting.* Structure of *Xb130* gene (5′ part), targeting construct, targeted allele and final knockout allele are shown (Empty Boxes: exons; S: SacI; B: BamHI, Filled boxes: Sites for Southern blot probes). Homologous recombination of ES cell genomic DNA with targeting construct inserts Loxp and Frt sites flanking an 879 bp region including Exon 7 and a floxed neomycin cassette. The deletion of Exon 7 causes a frame shift mutation and translation termination in Exon 8. ***B. Southern blotting with 3′ probe.*** Mice were derived from *Xb130^+/−^* progeny. ***C. Western blotting.*** Protein lysates were extracted from spleen and lung tissues of *Xb130^+/+^*, *Xb130^+/−^* and *Xb130^−/−^* mice that were genotyped by Southern blotting. ***D. PCR based genotyping***. Arrows indicate the location of primers used for PCR amplification. PCR product amplified from F1 and R1 in WT allele was undetectable due to competition from short product F2/R1. ***E. RT-PCR.*** The exons of RT-PCR products from WT (a) and knockout (b and c) mice are indicated based on sequencing data. Red stars indicate induced in-frame stop codons. ***F. Predicted Xb130 knockout allele based on RT-PCR and genomic PCR data.*** The primers used for long PCR and the amplicon are indicated as arrows and green line respectively.

A pSpuc plasmid was used to make the targeting construct to delete Exon 7 of the *Xb130* gene (gene ID. 226250). A 879 bp fragment, which includes the 3′ part of Intron 6, Exon 7 and 5′ part of Intron 7, was inserted between a loxp and a frt site flanking 5′ of neomycin cassette. A 5.5 kb long arm extending from 3′ of Intron 5 to 5′ of Intron 6 was cloned into the upstream segment of the loxp-879 bp fragment-frt-loxp. A 4.0 kb short arm containing 3′ of Intron 7 was inserted downstream of frt-loxp flanking 3′ of neomycin cassette (Fig. 1A).

#### Generation of targeted ES cells and *Xb130*-null mice

Embryonic stem cells from 129/Ola mice were used for gene targeting. G418+ clones were screened by Southern blotting. Targeted clones were treated with Flp enzyme to remove neomycin cassette. Selected ES clones were injected into blastocysts of peudopregnant C57BL/6 female mice to generate *Xb130^+/fl^* mice in 129/Ola B6 background. F1 *Xb130^+/fl^* mice were intercrossed with mice carrying Deleter-cre transgene. Ubiquitous Deleter-cre expression results in X*b130^+/−^* progeny that were confirmed by Southern blotting. *Xb130^+/−^* mice were backcrossed to a C57BL/6 background for more than 10 generations.

#### Genotyping

Characterization of ES clones and genotyping of littermates from *Xb130^+/−^* progeny were performed by Southern blotting. Genomic DNA was extracted from ES cells or mouse-tail and digested with BamHI. A 3′ probe corresponded to Exon 8 was used for hybridization. Probe was labelled with digoxigenin by PCR using PCR DIG Probe Synthesis Kit (Roche Applied Science, Indianapolis, IN). Hybridization was detected using Anti-Digoxigenin-Alkaline Phosphatase and CPD-star (Roche Applied Science). A band of 9 kb or 6.8 kb was detected in WT allele or floxed allele, respectively. A band of 8 kb was detected in KO allele. To confirm the presence of WT allele upstream of targeting vector sequence in targeting site, SacI digested mouse genomic DNA was hybridized with 5′ probe that includes the first 544 bp of 5′ of Intron 5. A 7.5 kb band was predicted in both WT and KO allele.

PCR genotyping was performed with three primers, including two common primers F1∶5′ CCTCTGCCGAAAACTCATAC 3′ and R1∶5′ ACAGCCGCTGTTGGTGGG 3′; and a WT allele-specific primer F2∶5′ GGGTAATTAACACCTGCTCAAAT 3′. The Amplicon from WT allele has a size of 748 bp, whereas the amplicon from the knockout allele has a size of 493 bp.

*RT-PCR*. Total RNA was extracted from lung tissues using Trizol reagent (Invitrogen, Carlsbad, CA). cDNA was synthesized using SuperScript first-Strand Synthesis for RT-PCR Kit (Invitrogen). RT-PCR was performed with primers E5F (5′ CGTGCTGAAGAAGAGC 3′) and E8R (5′ ATTGATGGATCTGTCAAATGG 3′), which correspond to the sequence of Exon 5 and antisense sequence of Exon 8. PCR products were purified and analyzed by sequencing.

#### Expand long genomic PCR amplification

Genomic long PCR product was amplified by Forward Primer GT-F1 (5′ CCTCTGCCGAAAACTCATAC 3′) and Reverse primer Intron7-R1 (5′ GAAACCCAAATACAATTTGTCTAGGCTGTAG 3′) using Expand Long Template PCR System (Roche Applied Science). PCR products were cloned into pBluescript SK^+^ vector and sequenced.

#### Western Blotting

For detection of XB130 protein, mouse tissues were homogenized using Tissuelyser II (Qiagen, Valencia, CA) in RIPA lysis buffer (150 mM NaCl, 1.0% NP-40, 0.5% DOC, 0.1% SDS, 50 mM Tris, pH 8.0) with a mixture of protease inhibitor cocktail (Roche Applied Science). After homogenization, tissue lysate were then maintained using constant agitation for 2 h at 4°C. The lysate was centrifuged for 20 min at 12,000 rpm at 4°C, and protein concentration was measured by BCA assay (Thermo Scientific, Waltham, MA). Proteins were denatured in Laemmli buffer and 10 µg of protein samples were subjected to SDS-PAGE and immunoblotting as previously described [13], [17]. Homemade rabbit polyclonal anti-XB130 antibody 92 was used for immunoblotting.

### Physiological assessment of *Xb130*-null mice

This study protocol was approved by the Toronto General Research Institute Animal Care and Use Committee (Permit Number: AUP1608.2). All animals received care in compliance with the Principles of Laboratory Animal Care formulated by the National Society for Medical Research, the Guide for the Care and Use of Laboratory Animals (National Institutes of Health publication No. 86–23, revised 1996), and the Guide to the Care and Use of Experimental Animals formulated by the Canadian Council on Animal Care. All surgery was performed under ketamine and xylazine anesthesia, and all efforts were made to minimize suffering. For animal sacrifice, all mice were anesthetized with isofurane first, and then sacrificed by cervical dislocation. For the physiological assessment, 4 mice were used for each genotype. For subcutaneous isogenic tracheal transplantation model, 4–8 tracheas were evaluated for each group at a particular time point.

#### Body composition evaluation

The body composition of mice was determined using a PIXI-mus Small Animal Densitometer (LUNAR, Madison, WI). Mice were anesthetized with 2% isoflurane. X-ray images and analyses included the tail but excluded the head. Obtained bone variables included bone mineral density (BMD; grams/cm^2^), bone mineral content (BMC; grams), and total bone area (TBA; cm^2^). The obtained tissue variables included lean (grams), fat (grams) and the percentage of fat.

#### Hematology test, blood biochemistry analysis, urinalysis and glucose tolerance test

The hematology test, which included red blood cell count (RBC, 10^12^/L), hemoglobin (Hgb, g/L), hematocrit (HCT, L/L), mean corpuscular volume (MCV, fL), mean corpuscular hemoglobin (MCH, pg/cell), mean corpuscular hemoglobin concentration (MCHC, g/L), white blood cell count (WBC, 10^9^/L) and platelet count (PLT, 10^9^/L) were examined. Blood biochemistry tests included alanine aminotransferase (ALT; U/L), aspartate aminotransferase (AST; U/L), creatin kinase (CK; U/L), L-lactate dehydrogenase (LDH; U/L) and uric acid (UA; umol/L). Urine was collected from conscious restrained mice. Urinalysis, including glucose, protein and blood in the urine, was performed.

The glucose tolerance test was performed using One Touch Ultra Glucometer (LifeScan Canada Ltd., Burnaby, Canada). Briefly, a 0.5–1 mm length of mouse tail is cut off using a sharp razor blade. A small drop of blood is collected and placed on the glucometer test strip to measure the blood glucose value (in mmol/L). Prior to the test, Mice had unlimited access to water but were fasted 15 h. The body weight and blood glucose concentrations were determined before and 30, 45 and 60 min after intraperitoneal injection of 2 mg of glucose per gram of body weight.

#### Mouse electrocardiography (ECG)

The mouse is anesthetized with 2% isoflurane in 700 ml O_2_/minute via facemask. Rectal temperature is maintained within 37–38°C using a heat pad and heat lamp. The lead II ECG is recorded from needle electrodes inserted subcutaneously into the right forelimb and into each hind limb. The signal is acquired for about 1 minute using a digital acquisition and analysis system (Power Lab/4SP, AD Instruments) and analyzed using the SAECG (signal-averaged electrocardiogram) extension for Chart 4.2.3 software. Unusually shaped P, QRS, or “T” waves and time-varying phenomenon (e.g. irregularities in interval durations) were examined and ectopic or abnormal beats were noted. SAECG was obtained from the averaged signal of a representative 10–15 s segment of the recording.

### Subcutaneous isogenic tracheal transplantation model for air way injury and repair

Adult male C57BL/6 mice (22–26 weeks) with *Xb130* KO and their WT littermates were used. Whole tracheal segments harvested from either WT or KO mice were subcutaneously transplanted to age matched WT or KO recipients (WT to WT and KO to KO)[9]–[11]. At different time points after transplantation, recipients were sacrificed and tracheal grafts were harvested. Tracheal segments were divided into three parts: upper and middle segments were fixed with 10% buffered formalin for histological studies, while the lower segments were snap-frozen in liquid nitrogen for RNA extraction.

#### Histology, immunohisochemistary and immunofluorescence studies

Formalin-fixed tracheal grafts were embedded in paraffin wax, cut into 4 µm sections and stained with hemotoxylin and eosin (H&E). The slides were examined with an Axiovert 200 M microscope (Zeiss, Oberkochen, Germany), and images were captured via CoolSnap HQ camera (Roper, Ottobrunn, Germany). The area of tracheal epithelium and the length of basement membrane were measured using ImageJ (1.46r) (NIH, Bethesda, MD). The number of epithelial cells was determined by counting hematoxylin stained nuclei. The thickness of epithelium was calculated as the area of tracheal epithelium/the length of basement membrane. The cell density was calculated as the number of epithelial cells/100 µm basement membrane. For the evaluation of the epithelium thickness and cell density, 6 random sections from the upper and middle segments of each trachea were measured, and 4–8 tracheas were evaluated for each group at a particular time point.

Formalin-fixed and paraffin-embedded tracheal grafts tissues sections were deparaffined in xylene and rehydeated in a graded alcohol series and transferred to PBS. Standard immunohistochemistray was performed as previously reported [15]. Antigen retrieval was performed through boiling the tissue slide in 10 mmol/L citrate buffer (pH 6.0) for 20 min before cooling down to room temperature for 20 min. The slides were then incubated with 3% H_2_O_2_ for 30 min to block endogenous peroxidase activity. Non-specific binding was blocked using 5% goat serum applied for 60 min. Slides were incubated with polyclonal rabbit anti-Ki67 antibody (1∶200, Abcam, Cambridge, MA) overnight at 4°C, and then with the secondary antibody (biotinylated anti-rabbit IgG, 1∶200 in PBS) for1 h in a humidified chamber. Detection was made using a Vecstatin ABC kit (Vector Laboratories, Burlington, ON) with 3–3-diaminobenzidine as chromogen. Slides were counterstained with hematoxylin. Ki-67-stained cells were quantified in eight randomly selected fields at 200× magnification. The percentage of Ki-67+ staining cells was calculated [15].

For immunofluorescence staining, tissue slides were incubated with rabbit polyclonal antibody to Cytokeratin 5 (CK5) (1∶1000, Abcam) or with monoclonal XB130 antibody at room temperature for 60 min. Secondary antibodies were Alexa Fluor 555 Goat Anti-Rabbit IgG (H+L) and Alexa Fluor 555 Goat Anti-Mouse IgG (H+L) (1∶200, Invitrogen), respectively. Slides were counterstained with 4′, 6-diamidino-2-phenylindole (DAPI) (Invitrogen) and mounted with SlowFade. CK5+ staining was quantified using Image J (1.46r). Data are expressed as percent+staining/unit length [20].

#### TUNEL assay

Apoptosis was assessed by in situ terminal deoxynucleotidyl transferase-mediated dUTP nick end-labeling (TUNEL) staining (In Situ Cell Death Detection Kit, TMR red, Penzberg, Upper Bavaria, Germany) according to manufacturer’s instructions. DAPI was used for nuclear staining.

#### Quantitative RT-PCR

Total RNA was extracted using RNeasy kit (Qiagen, Duesseldorf, Germany). cDNA was synthesized from total RNA using MuLV Reverse Transcriptase (Invitrogen). Quantitative RT-PCR was performed using SYBR Green I Master PCR kit on LightCycler480 (Roche Diagnostics). Each assay included a standard curve of six serial dilutions and a no-template negative control. All assays were performed in triplicate. The primers used are shown in Table 1. The relative expression level of each target gene was calculated after normalization with housekeeping gene GAPDH.

**Table 1:** Primers of the selected genes for quantitative RT-PCR.

Genes	Forward primer	Reverse primer
*Trp63*	5′ TAC TGC CCC GAC CCT TAC AT 3′	5′ GCT GAG GAA CTC GCT TGT CTG 3′
*Ck5*	5′ TCT GCC ATC ACC CCA TCT GT 3′	5′ CCT CCG CCA GAA CTG TAG GA 3′
*Foxj1*	5′ CCC TGA CGA CGT GGA CTA TG 3′	5′ GCC GAC AGA GTG ATC TTG GT 3′
*Scgb1a1*	5′ ATG AAG ATC GCC ATC ACA ATC AC 3′	5′ GGA TGC CAC ATA ACC AGA CTC T 3′
*Ki67*	5′ CCC TGA CGA CGT GGA CTA TG 3′	5′ GCC GAC AGA GTG ATC TTG GT 3′
*Xb130*	5′ TCA GCA TCT CCA GAC 3′	5′GGC TGT TTC CTC TCT 3′
*Gapdh*	5′ ATG AAG ATC GCC ATC ACA ATC AC 3′	5′ GGA TGC CAC ATA ACC AGA CTC T 3′

### Statistical analysis

Statistical analyses were calculated using Student’s t-test or analysis of variance (ANOVA). Results were expressed as the mean ± standard error of the mean (SEM). Statistical analyses were performed using GraphPad Prism 5.01 (GraphPad, La Jolla, CA). P values less than 0.05 were considered statistically significant.

## Results

### Generation of *Xb130* KO mice

To determine the function of XB130 *in vivo*, we generated *Xb130* knockout mice using a conventional gene targeting strategy[21]–[23]. *Xb130* contains 21 exons and has three major putative alternative splicing transcript variants in the 5′ end. These transcript variants may encode proteins that differ in the N-termini with 843aa, 825aa, and 769aa (Gene ID: 226250, NCBI). We used a strategy to delete Exon 7, a common exon that exists in all three variants, to induce a stop codon in Exon 8 via frame shift mutation that resulted in a truncated open reading frame in all three putative transcript variants (Fig. 1A). The floxed *Xb130* allele in ES clones (data not shown) and Cre cleaved null allele in mice (Fig. 1B) were confirmed by Southern blotting. The depletion of XB130 protein in *Xb130^−/−^* mice was further confirmed by western blotting in the spleen and the lung (Fig. 1C).

Although Southern and western blotting confirmed the null allele of *Xb130^−/−^* mice, these mice displayed both WT and KO alleles in a PCR-based genotyping (Fig. 1D). To understand the conflict between PCR and Southern blotting genotyping, we checked the transcripts of *Xb130* null allele in *Xb130^−/−^* mice by RT-PCR. Interestingly, *Xb130^−/−^* mice expressed a longer transcript than WT (Fig. 1E). The sequence analysis of RT-PCR products showed that the Exon 7 sequence was not deleted in transcripts from *Xb130* null allele. Instead, Exon 6 and loxp sequence were inserted between Exon 7 and Exon 8. The insertion induced frame-shift mutations and caused translation termination at the breakpoint (Fig. 1E). The predicted proteins translated from truncated open reading frames didn’t contain any conserved domains that are important in XB130 function. RT-PCR data indicated a one-end gene targeting event that resulted from a homologous recombination event at 3′ short homologous arm of the targeting construct accompanied by a non-homologous end joining event at the 5′ homologous arm (Fig. 1F). Long-range genomic PCR amplification & sequencing verified the homologous recombination event at 3′ arm (Fig. 1F and data not shown). Southern blotting using 5′ probe supported the notion of a non-homologous end join event at the 5′ arm (Fig. 1F and data not shown). Since it is not a true allele replacement, a PCR strategy did not apply to *Xb130^−/−^* mice genotyping.

### Adult *Xb130* KO mice did not show phenotypic differences

**Figure 2:**
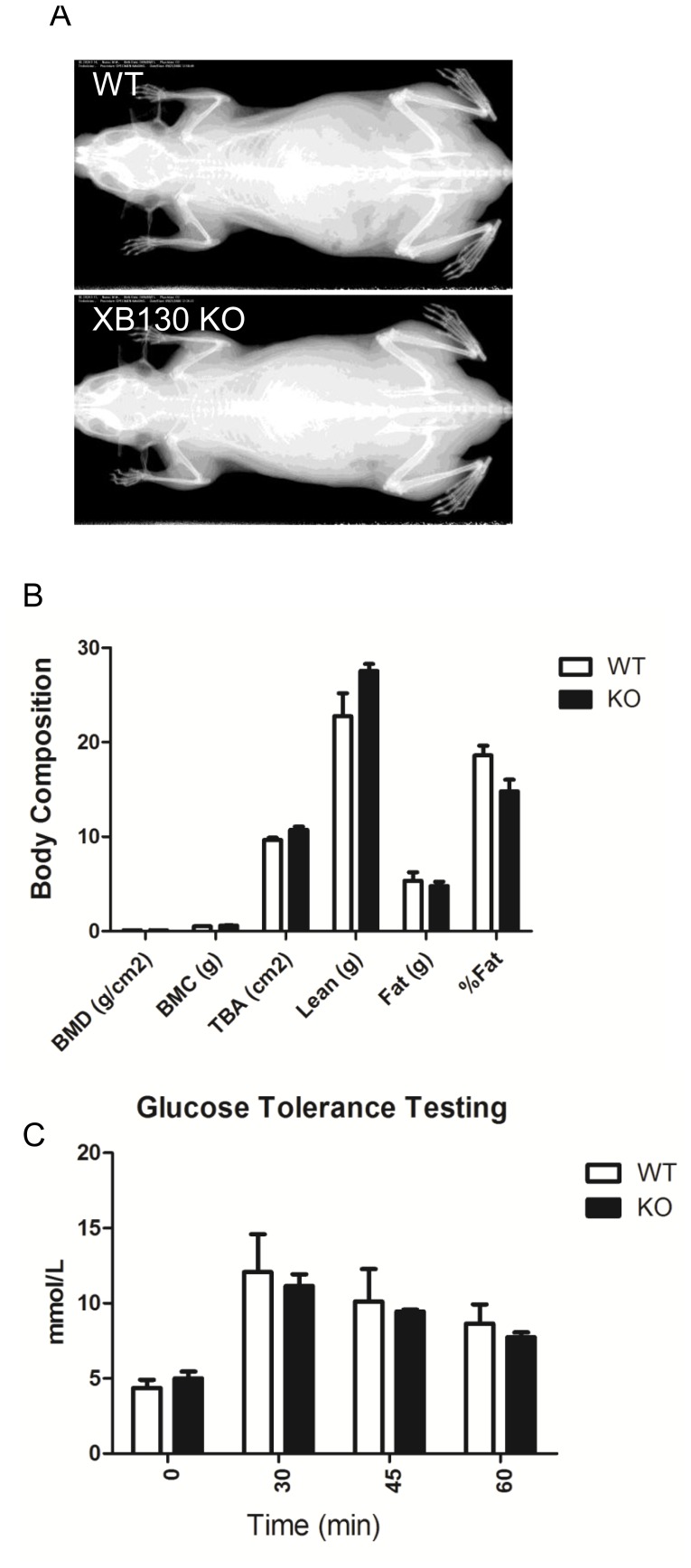
X-ray, body composition analysis and glucose tolerance testing for WT and KO mice. In adult mice, no significant differences were found between WT and KO mice on X-ray (A), body composition (B) and ECG (C).

*Xb130* knockout mice appeared normal at birth and showed normal life span. A series of physiological tests, including skeletal X-ray (Fig. 2A), body composition (Fig. 2B), ECG, hematology, blood biochemistry, urinalysis and glucose tolerance test (Fig. 2C), were performed. However, there were no obvious phenotypic differences between KO mice and their WT littermates (Fig. 2, Table 2–4).

**Table 2:** No significant differences of hematological parameters between *Xb130 KO* and WT mice.

Groups	RBC (10^12^/L)	Hgb (g/L)	HCT (L/L)	MCV (fL)	MCH (pg/cell)	MCHC (g/L)	WBC (10^9^/L)	PLT (10^9^/L)
WT	9.94±0.16	153.25±2.43	0.48±0.01	47.95±0.79	15.43±0.23	321.75±1.70	3.28±0.25	1562.50±211.86
*Xb130 KO*	10.11±0.23	160.67±1.67	0.50±0.01	49.10±1.44	15.90±0.47	324.33±0.33	7.63±2.53	1315.33±83.13
p	0.55	0.07	0.17	0.48	0.36	0.26	0.10	0.39

RBC: red blood cell count; Hgb: hemoglobin; HCT: hematocrit; MCV: mean corpuscular volume; MCH: MCHC: mean corpuscular hemoglobin concentration; WBC: white blood cell count; PLT: platelet count.

**Table 3:** No significant differences of blood biochemistry between *Xb130 KO* and WT mice.

Groups	ALT (U/L)	AST (U/L)	CK(U/L)	LDH (U/L)	UA (umol/L)
WT	44.00±6.21	56.00±4.30	196.00±52.21	565.00±95.42	13.00±2.00
*Xb130 KO*	37.00±4.64	60.00±8.80	327.00±171.34	464.00±73.15	10.00±2.03
p	0.39	0.69	0.49	0.43	0.32

ALT: alanine aminotransferase; AST: aspartate aminotransferase; CK: creatin kinase; LDH: L-lactate dehydrogenase; UA: uric acid.

**Table 4:** No significant differences of ECG parameters between *Xb130 KO* and WT mice.

Groups	Rate (bpm)	PR (ms)	QRS (ms)	QT (ms)	Qtmax (ms)	QTc (ms)	QRSH (mV)	PH (mV)	Cycle
WT	486.7±13.2	37.7±1.44	9.50±0.65	22.00±0.57	10.75±0.75	19.80±0.40	1.55±0.10	0.09±0.01	126.7±3.04
*Xb130 KO*	451.9±33.6	40.3±0.33	10.67±0.33	23.67±0.33	11.67±0.67	20.50±0.76	1.65±0.13	0.07±0.01	127.0±5.57
*p-*Value	0.29	0.13	0.35	0.06	0.61	0.44	0.50	0.17	0.89

Rate (bpm): The reciprocal of the average RR interval between valid beats; PR (ms) : Time interval between the first P wave marker and the first QRS marker (P1 to Q1); QRS (ms): Time interval between the QRS wave markers (Q1 to Q2); QT (ms): Interval between the first QRS marker and the second “T” wave marker (Q1 to T2); QTmax (ms): Time interval between the first QRS marker and the time of the maximal amplitude (positive or negative) of the “T” wave (Q1 to Tmax); QTc (ms): The QT interval ‘corrected’ for heart rate according to the formula QTc = QT/(RR/100)0.5; QRSH(mV): The maximum value occurring between the QRS markers, minus the minimum value; PH (mV): The maximum value occurring between the P wave markers, minus the minimum value; Cycles: The number of cardiac cycles included in the averaged ECG waveform.

### Histological change of tracheal epithelium in *Xb130* KO mice

**Figure 3:**
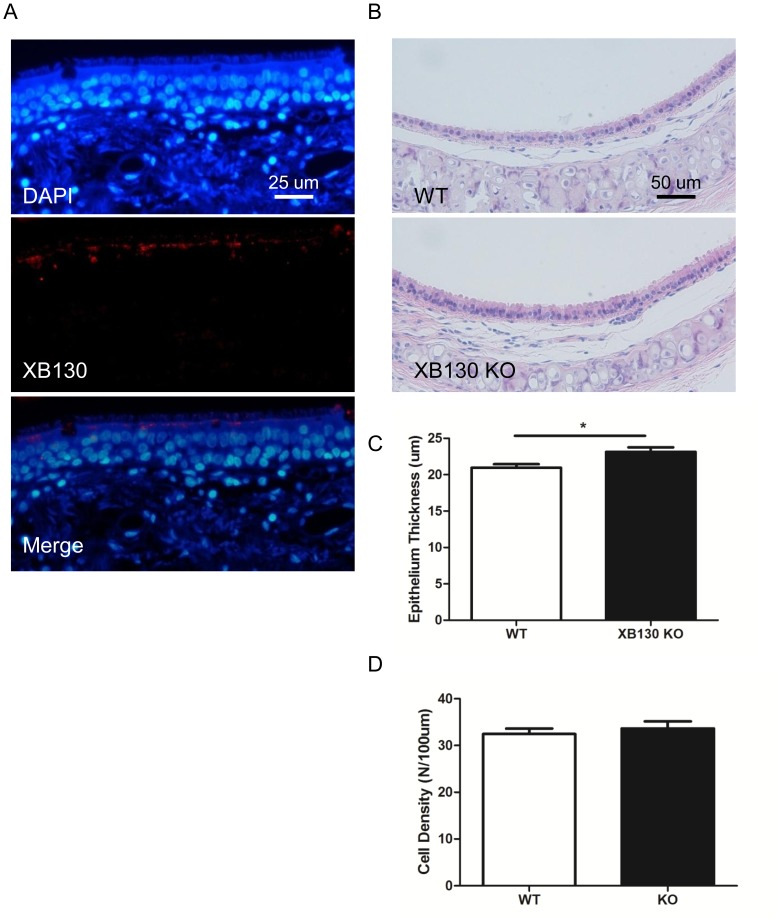
XB130 is expressed at the apical site of ciliated epithelial cells in human trachea, and thicker tracheal epithelium is seen in *Xb130* KO mice. In human tracheal epithelium, XB130 is located in the cytoplasm near the apical sides. There is no XB130 expression in the basal cells (A). In mice, the tracheal epithelium in *Xb130* KO mice is thicker compared with WT mice (B and C), but there is no difference in the epithelial cell density (D).

XB130 has been found in epithelial cells of thyroid [15], lung [13], esophagus [19], stomach [18] and other human organs. To explore the role of XB130 in tracheal epithelium, the expression and location of XB130 was detected in human tracheal epithelium using immunofluorescence staining. XB130 was strongly expressed in the cytoplasm of ciliated cells and located on the apical side under the microvilli (Fig. 3A). This particular distribution of XB130 suggests that it may be related to secretion and microvilli function.

We then examined the morphology of tracheal epithelium in mice. There was no apparent difference in tracheal epithelium between WT and *Xb130* KO mice (Fig. 3B). But, the tracheal epithelium was significantly more thick in *Xb130* KO mice than that found in WT mice (Fig. 3C). There was no difference in terms of cell density (the number of cells per µm) between WT and *Xb130* KO mice (Fig. 3D), suggesting an elongation of epithelial cells from apical to basal-lateral direction.

### *XB130* deficiency did not affect tracheal transplant-related epithelial injury

**Figure 4:**
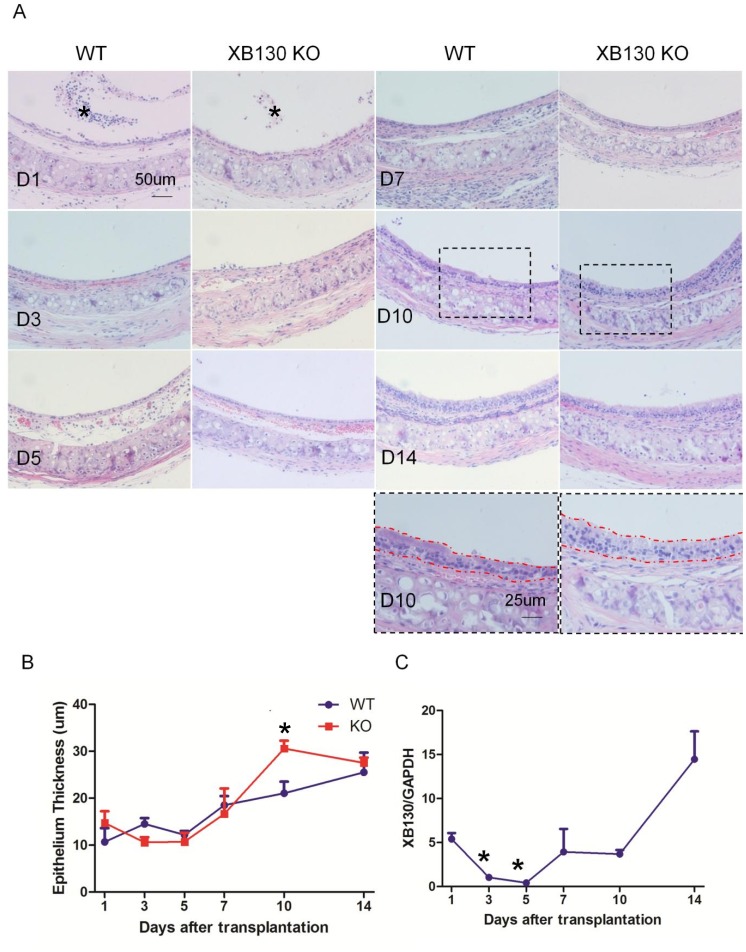
A transient tracheal epithelium hyperplasia is seen in *Xb130* KO mice during airway repair after isogenic tracheal subcutaneous transplantation. A dynamic airway epithelium injury and repair process is observed in both WT and *Xb130* KO mice (A). One day after tracheal transplantation, many epithelial cells detached from the basement membrane (D1, * the detached epithelial cells). On Day 3, survived epithelial cells became flat and elongated to cover the basement membrane (D3). From Day 5 to Day 7, epithelium was repopulated with cuboidal shaped cells (D5, D7). The epithelial cells were further differentiated towards pseudo-stratified layer (D10, D14). Severe epithelial hyperplasia was noted in KO mice in comparison with WT mice at Day 10 (A and B). After transplantation, XB130 mRNA levels were reduced at Day 3 and Day 5, and then increased afterwards (C).

**Figure 5:**
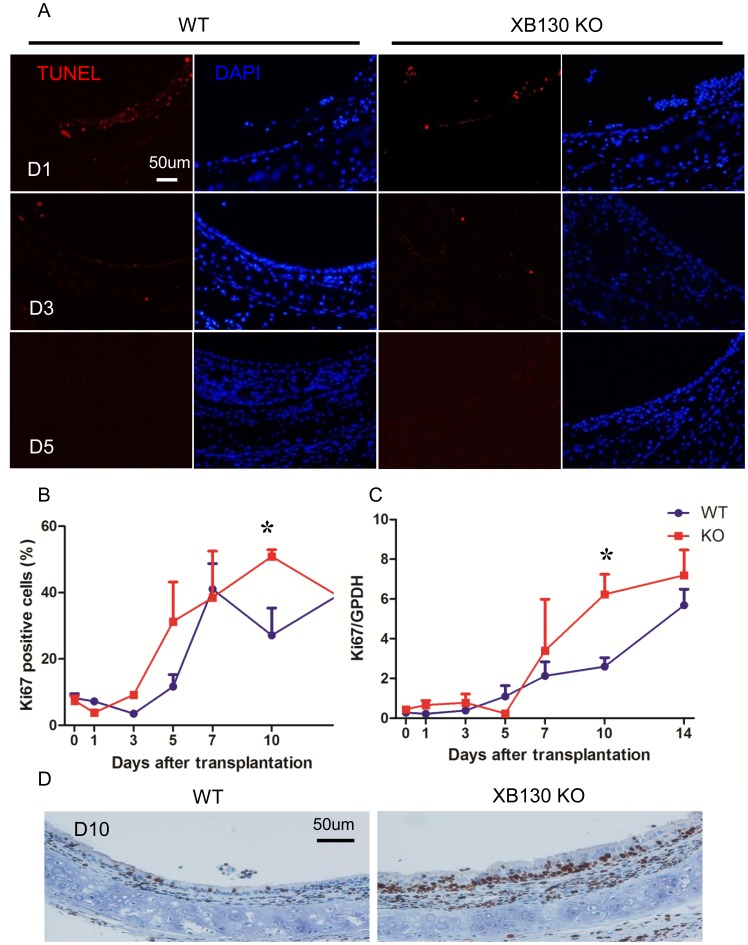
*Xb130* KO tracheal epithelium exhibits comparable cell apoptosis but higher cell proliferation at Day 10 after isogenic tracheal subcutaneous transplantation. In both group, comparable tracheal epithelium apoptosis was observed at an early stage after transplantation (D1 and D3). After Day 5, no apoptosis cells were observed. After tracheal transplantation, the proliferation in both groups was reduced and then increased gradually (B and C). At Day 10 after transplantation, cell proliferation is significantly higher in *Xb130* KO group as determined by Ki67 immunohistochemistry staining (B) and Ki67 mRNA expression (C). Representative Ki67 staining also showed increased thickening of connective tissue layer between the epithelium and cartilage, and fibroblast proliferation (D).

In order to explore the role of XB130 in airway injury, repair and regeneration, a mouse heterotypic tracheal transplantation model was used. In this model, the grafts experienced an unavoidable ischemia-reperfusion process, which induced severe epithelial damage immediately after transplantation. As shown in Figure 4A, one day after transplantation, many epithelial cells detached from the basement membrane and were found in the airway lumen (Fig. 4A–D1, indicated with *). TUNEL staining revealed that most of the detached cells were apoptotic (Fig. 5A–D1). On Day 3, survived cells became flat and elongated, covering the surface of the basement membrane (Fig. 4A–D3). There were no significant differences between the WT and KO groups, in terms of histology (Fig. 4A) and the number of apoptotic cells (Fig. 5A, and data not shown).

### Tracheal epithelial hyperplasia in *Xb130* KO mice during airway repair

With revascularization of the grafts, a spontaneous repair process occurred. From Day 5 to Day 7, epithelium was repopulated with cuboidal shaped cells (Fig. 4A, D5–D7). From Day 10 to Day 14, epithelial cells were further differentiated towards a pseudo-stratified morphology (Fig. 4A, D10–D14). The overall histological features of epithelium were similar between WT and KO group. Detailed morphometric analysis shows that the thickness of epithelial layer increased gradually. On Day 10, epithelial thickness in the WT group was 21.06±2.46 µm (Fig. 4B), comparable with 20.96±0.49 µm in normal trachea (Fig. 3C). By contrast, on Day 10, the epithelial thickness in KO mice was 30.60±1.66 µm, significantly higher than that in the WT mice (21.06±2.46 µm, Fig. 4B), and also much higher than that in the normal trachea of *Xb130* KO mice (23.13±0.64 µm, Fig. 3C). However, epithelial thickness was reduced back to almost “normal levels” on Day 14, indicating a transient hypertrophy/hyperplasia in the KO mice group.

After transplantation, *Xb130* mRNA expression experienced a dynamic change in the trachea of WT mice. Expression levels of *Xb130* mRNA were significantly reduced at Day 3 and Day 5, recovered to the basal level at Day 7 and Day 10, and became significantly elevated at Day 14 (Fig. 4C).

The expression of Ki67 protein and mRNA levels were used as markers for cell proliferation. Ki67+ cells (Fig. 5B), determined with immunohistochemisty staining, and *Ki67* mRNA levels (Fig. 5C), evaluated with quantitative RT-PCR, were very low between Day 0 and Day 3, before becoming significantly elevated at Day 5 and remaining at high levels (Fig. 5B and 5C). At Day 10, the number of Ki67+ cells (Fig. 5B) and *Ki67* mRNA level (Fig. 5C) in the KO group were significantly higher than that in the WT group. On Day 10, we also noticed that there were much thicker connective tissue proliferating fibroblasts between epithelium and tracheal cartilages in the *Xb130* KO mice compared with WT mice (Fig. 5D). Taken together, these results suggest that in the absence of XB130, epithelial repair was associated with a transient hyperplasia, which delays the recovery of epithelium after injury.

### Increased CK5+ airway basal cells in *Xb130* KO mice during repair

**Figure 6:**
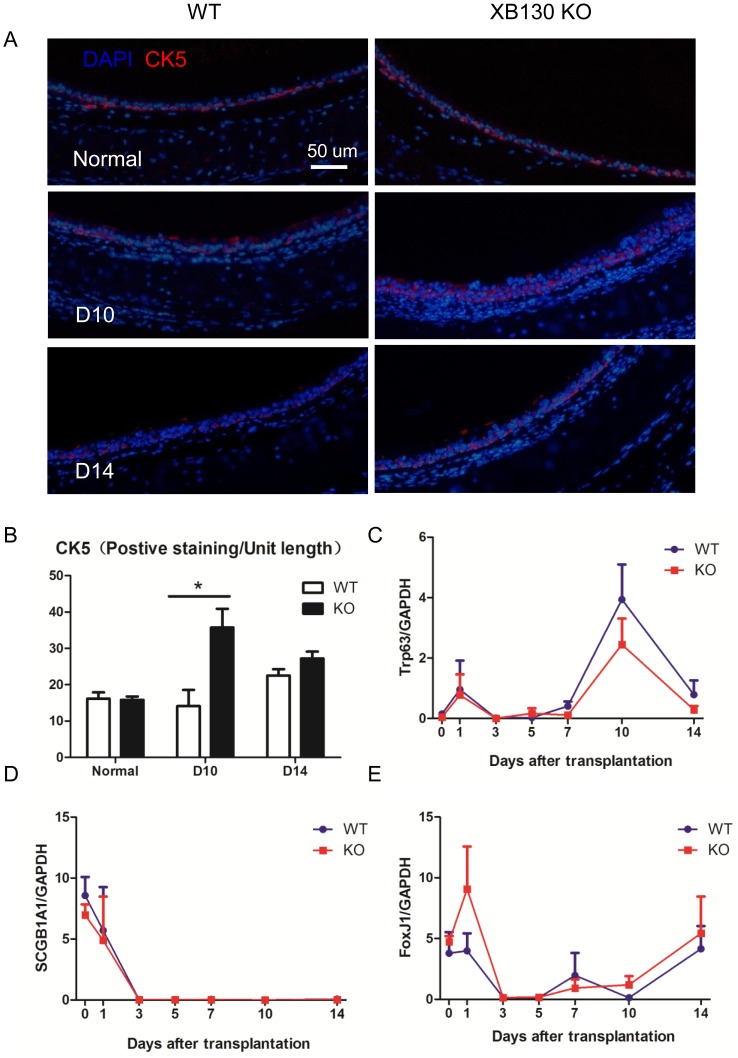
Increased CK5+ airway basal cells are noted at Day 10 after tracheal transplantation. Basal cells were determined by immunofluorescent staining for Cytokeratin 5 (CK5). More CK5+ cells were found in the KO group at D10 after transplantation (A and B). The mRNA levels of *Trp63* (C, basal cell marker), *Scgb1A1* (D, Clara cell marker) and *FoxJ1* (E, ciliated cell marker) were examined with RT-PCR. Dynamic changes of mRNA levels were noted during airway injury and repair. However, there was no difference between WT and KO groups.

In the large airway, cytokeratin-5 (*CK5*)+ basal cells are identified as local progenitor cells[3], [24]–[28]. In this trachea transplantation model, it has been shown that CK5+ basal cells are the major survivors after injury and can further proliferate and differentiate into other types of epithelial cells to repair the epithelium [11]. We suspected that the increased cell proliferation (Ki67+ cells) at Day 10 in *Xb130* KO mice should be at least partially due to increased CK5+ basal cells. CK5+ basal cells were studied using immunofluorescence staining (Fig. 6A). There was no difference in CK5+ cells in normal tracheal epithelium between WT and KO mice. However, the number of CK5+ cells at Day 10 was significantly higher in *Xb130* KO mice than that in WT mice (Fig. 6B). In addition to CK5, CK14 and Trp63 (p63) are also markers of basal cells [29]. The Foxj1 is a forkhead domain transcription factor required for late stages of ciliogenesis [30], and is a marker of ciliated cells [10], [11]. Scgb1A1 (i.e., CCSP) is a marker of Clara cells[31]–[34]. The expressions of these genes were examined using RT-PCR. The mRNA levels of *CK5* and *CK14* fluctuated after tracheal transplantation (data not shown). *Trp63* mRNA levels were highly expressed at Day 10 (Fig. 6C). Clara cell marker *Scgb1A1* levels dropped after the tracheal transplantation and did not recover during the study period (Fig. 6D). The expression of *Foxj1*, a marker of ciliated airway epithelial cells, was reduced to very low levels at Day 3, and then gradually returned to basal levels (Fig. 6E). There were no significant differences between WT and *Xb130* KO mice at different time points (Fig. 6C, 6D and 6E).

## Discussion

As an adaptor protein, XB130 has been shown to be important in signal transduction and in the regulation of cell survival, proliferation and migration, which are important for tissue or organ repair[16], [35]–[38]. Therefore, we expected that transplant-related ischemic injury may be more severe in *Xb130* KO mice due to increased apoptosis, and the repair process may be slowed down due to less cell migration and proliferation. To our surprise, the severity of epithelial injury, apoptosis, cell spreading, and expression of cell type markers were similar between KO and WT mice at most time points studied. However, the significant thickness of epithelial layers in normal trachea, and increased hyperplasia (thickening of epithelial layer and more Ki67+ proliferating cells) and CK5+ basal cells at Day 10 after transplantation in KO mice strongly suggests that XB130 in the airway epithelium plays a role in determining the differentiation of epithelial cells.

We detected the expression and location of XB130 in human tracheal epithelium. XB130 is mainly located in the cytoplasm and enriched near the apical site of ciliated cells, especially near the bottom of the cilia. This suggests that at the apical site of the plasma membrane, XB130 may be involved in the function of microvilli and other cellular functions, such as secretion, ion transportation, and absorption. The anti-human XB130 monoclonal antibody has been used to study expression of XB130 in human tissues [15], [18], [19], [39]. However, it does not cross-react with mouse XB130 for staining purposes. We also tested several commercially available anti-human XB130 antibodies, but none showed good specific staining in mouse tissues (data not shown). This is an observed limitation of our study.

In this study, we generated *Xb130* KO mice. Through detailed evaluation of the different physiological tests, no abnormal parameters were found in *Xb130* KO mice compared with WT mice. These results indicate that the function of XB130 can be compensated in young adult mice. Comparing the morphology of tracheal epithelium between XB130 WT and KO mice, we found that the epithelial thickness in *Xb130* KO mice was significantly higher, which further suggests that in the absence of XB130, the differentiation of tracheal epithelium is affected. Rigorous uniform sampling designs of stereology ensures unbiased estimation of number, length, surface area and volume [40]–[41] should be considered in the future studies, to be more precisely define the role of XB130 in the epithelial differentiation. The physiological functions of tracheal epithelium in these KO mice should also be further studied, for instance, using air-liquid interface culture system.

In order to further study the effect of XB130 deficiency on tracheal epithelial injury, repair and regeneration, a subcutaneous isogenic tracheal transplantation model was used. In both *Xb130* WT and KO mice, a reproducible epithelium injury, repair and regeneration process was observed. During airway repair, the thickness of the epithelium gradually increased towards the basal levels found in normal trachea. Interestingly, the thickness of epithelium in *Xb130* KO mice was significantly higher than that of the WT group at Day 10. The thicker epithelium is not only the reflection of hypertrophy (enlargement of cell size), but also hyperplasia (increased cell number), as indicated by an increase in proliferating Ki67+ cells. Furthermore, we noted a higher number of CK5+ basal cells at Day 10. Since CK5+ cells are local progenitor cells of ciliated airway epithelial cells[25]–[27], and XB130 is expressed only in ciliated epithelial cells in normal trachea, this finding suggest a delay from undifferentiated basal (CK5+) cells to well-differentiated ciliated cells in *Xb130* KO mice. These results suggest that during the repair process, the absence of XB130 may affect the differentiation of epithelium. Considering the relatively thicker epithelium seen in normal trachea, we speculate that XB130 is indeed required for the maintenance of normal structure and function in the tracheal epithelium.

In the adult mouse trachea, basal cells constitute approximately 30% of the total epithelial cells [25], [27]. Basal cells in the pseudostratified epithelium of the mouse trachea express CK5 and CK14, as well as Trp63. The postnatal development of basal cells is dependent on Trp63, and *Trp63*-null mice lack basal cells [29]. In the present study, the expression of *Trp63* mRNA showed the highest expression on Day 10, coincidently alongside the highest cell proliferation (Ki67+ staining) and CK5+ staining. However, we did not find any difference in *Trp63* mRNA levels between WT and KO group, whereas CK5 staining showed significantly higher levels in the trachea of *Xb130* mice. Using lineage tracing, it has been shown that CK5 is essential for basal cells to generate differentiated cells during postnatal growth and in the adult during both steady state and epithelial repair [27]. Even though both Trp63 and CK5 are markers for basal cells, there is differential expression of these proteins in airway basal cells [3]. During airway repair, Trp63 and CK5 may represent different sub-populations of basal cells. Moreover, expression of CK5 mRNA and protein may be regulated differently.

At Day 10 after transplantation, we noticed that the connective tissue and fibroblast layer between epithelium and tracheal cartilages were much thicker in *Xb130* KO mice compared with WT mice. Many Ki67+ proliferative cells were observed in this layer in *Xb130* KO mice. Identifying these cells and deposition of matrix proteins should be considered. It has been reported that in the small airway, cross-talk between parabronchial smooth muscle cells and airway epithelial stem cells is involved in epithelial cell proliferation and differentiation after injury [42]. Similar cross-talk may also exist between the tracheal epithelial cells and mesenchymal cells, and XB130 may be involved in this process.

In summary, XB130, located on the apical site of ciliated epithelial cells in the trachea, may participate in maintaining differentiation and function in these cells. During the repair process, a lack of XB130 may influence the differentiation of proliferating basal cells to differentiated ciliated epithelial cells.
